# Molecular classification and biomarkers of clinical outcome in breast ductal carcinoma *in situ*: Analysis of TBCRC 038 and RAHBT cohorts

**DOI:** 10.1016/j.ccell.2023.06.002

**Published:** 2023-07-10

**Authors:** Siri H. Strand, Belén Rivero-Gutiérrez, Kathleen E. Houlahan, Jose A. Seoane, Lorraine M. King, Tyler Risom, Lunden A. Simpson, Sujay Vennam, Aziz Khan, Luis Cisneros, Timothy Hardman, Bryan Harmon, Fergus Couch, Kristalyn Gallagher, Mark Kilgore, Shi We, Angela DeMichele, Tari King, Priscilla F. McAuliffe, Julie Nangia, Joanna Lee, Jennifer Tseng, Anna Maria Storniolo, Alastair M. Thompson, Gaorav P. Gupta, Robyn Burns, Deborah J. Veis, Katherine DeSchryver, Chunfang Zhu, Magdalena Matusiak, Jason Wang, Shirley X. Zhu, Jen Tappenden, Daisy Yi Ding, Dadong Zhang, Jingqin Luo, Shu Jiang, Sushama Varma, Lauren Anderson, Cody Straub, Sucheta Srivastava, Christina Curtis, Rob Tibshirani, Robert Michael Angelo, Allison Hall, Kouros Owzar, Kornelia Polyak, Carlo Maley, Jeffrey R. Marks, Graham A. Colditz, E. Shelley Hwang, Robert B. West

(Cancer Cell *40*, 1521–1536.e1–e7; December 12, 2022)

In the originally published version of this paper, some errors were introduced during the final preparation of Table 1. First, for the RAHBT cohort, the values for “Lumpectomy + radiation” and “Lumpectomy − radiation” were transposed. Second, in the RAHBT “DCIS with invasive recurrence” group, the number of samples with grade 3 DCIS should be n = 3, not 2 as listed. Third, for the RAHBT “DCIS without recurrence” group, there should be n = 53 ER+, not 55 as listed; and n = 15 ER−, not 13 as listed.

Table 1 has been corrected in the original article online. The authors apologize for any confusion these errors might have caused. Since these errors only occurred in Table 1, none of the analyses or the conclusions of the paper are affected.

